# Identification, characterization, and expansion of innate lymphoid cells derived from canine peripheral blood mononuclear cells

**DOI:** 10.3389/fimmu.2026.1879697

**Published:** 2026-07-15

**Authors:** Yeganeh Mehrani, Lily Chan, Charly McKenna, Mikaela Eng-Toogood, Fernando de Paula Freitas, Emma Vanderboon, J. Paul Woods, Byram W. Bridle, Khalil Karimi

**Affiliations:** 1 Department of Pathobiology, Ontario Veterinary College, University of Guelph, Guelph, ON, Canada; 2 Department of Clinical Studies, Ontario Veterinary College, University of Guelph, Guelph, ON, Canada

**Keywords:** canine immunology, canine innate lymphoid cells, comparative immunology, flow cytometry, peripheral blood mononuclear cells, transcription factors

## Abstract

Innate lymphoid cells (ILCs), a family of innate immune cells of lymphoid origin, serve as first responders to tissue damage and inflammation. They play a crucial role in defending mammals against tumor growth. ILCs can migrate between organs, move within tissues, and replenish from the blood during inflammation. Although much is known about ILCs in humans and mice, their properties in dogs are still largely unclear. To characterize canine ILCs, peripheral blood mononuclear cells (PBMCs) were isolated and cultured to expand ILCs. PBMCs were stimulated with recombinant human interleukin-2 (rhIL-2) and recombinant canine interleukin-7 (rcIL-7) for ILC1s expansion, rcIL-33 for ILC2s, or rh/rcIL-23 for ILC3s. Flow cytometry identified ILCs in PBMCs and cultures. We demonstrated that fresh PBMCs contained distinct populations of ILC1s, ILC2s, and ILC3s, characterized by the expression of cell surface markers (Lin^−^, CD45^+^, CD127^+^) and the subset-specific transcription factors T-bet^+^ (ILC1s), GATA-3^+^ (ILC2s), and RORγt^+^ (ILC3s). Following the expansion on days 0, 7, 14, and 21, the purity of each ILC subset increased, peaking on day 14. The expanded ILCs upregulated subset-specific transcription factors, indicating differentiation and enrichment. These findings provide a reliable method to isolate, characterize, and expand canine ILCs from blood, useful for research on their roles in health, disease, and potential therapies. They also offer insights into human cancer research, as dogs develop cancers similarly to humans.

## 1 Introduction

ILCs are heterogeneous members of the lymphoid lineage and components of the innate immune system that play critical roles in tissue homeostasis and inflammation (1). They serve as the innate counterparts of T lymphocytes and mirror key aspects of their phenotype and function (2). Therefore, like CD4+ T cells, Th1, Th2, and Th17, ILCs are currently classified into three major subsets based on the expression of surface markers, transcription factors, and effector cytokines (3): ILC1 (including both NK cells and non-cytotoxic or non-NK ILC1) produce the Th1-type cytokine IFN-γ and are mainly characterized by the transcription factor T-bet, with Eomes expressed in NK cells. ILC2s generate Th2-type cytokines IL-4, IL-5, and/or IL-13 and depend on the lineage-defining transcription factor GATA-3. ILC3, which includes two subpopulations (natural cytotoxicity receptor [NCR]+ and NCR- ILC3), secrete Th17-type cytokines IL-17 and/or IL-22 (4–6), and are defined by the expression of RORγt.

CD127, also known as the interleukin-7 receptor alpha chain (IL-7Rα), is a key component of the receptor complex that mediates IL-7 signaling, which is essential for the development, survival, and maintenance of lymphoid cells. In both humans and mice, CD127 is widely used as a defining marker to identify ILCs and to distinguish these populations from other immune cell subsets. Therefore, CD127 was included in our gating strategy as a core marker for identifying canine ILC populations.

ILCs quickly respond to cytokines released by other cells, producing and releasing distinct cytokines. For instance, in response to IL-12, IL-15, and IL-18 from dendritic cells (DC), ILC1s release IFN-γ, while ILC2s secrete IL-4, IL-5, IL-9, IL-13, and amphiregulin in response to IL-25, IL-33, and thymic stromal lymphopoietin from epithelial cells and tissue-resident immune cells. ILC3s produce IL-17 and IL-22 in response to IL-23, IL-1β, and IL-1α from macrophages and DCs (7, 8).

Given the expression of specific cell-surface and/or intracellular molecules by different ILC subsets, flow cytometry is essential for studying ILCs. Human ILCs are identified based on their surface marker expression and the transcription factors required for their development or function, such as ILC1, ILC2, and ILC3, defined respectively by IFN−γ production and T−bet dependence, type−2 cytokine production with GATA−3 and RORα, and IL−17/IL−22 production driven by RORγt and AHR (9, 10).

Accordingly, mouse ILCs are also classified as i. NK cells (CD3^-^ CD49b^+^ NKp46^+^ T-bet^high^ Eomes^high^), ii. ILC1 (lineage-specific markers negative [Lin-] CD49b^+^ NKp46^+^ CD127^+^ T-bet^high^), iii. ILC2 (Lin^-^ CD90.2^+^ T1/ST2^+^ ICOS^+^ CD25^+^GATA3^high^), and iv. NCR- ILC3 (Lin^-^ NKp46^-^ T-bet^+/-^ RORγt^+^), and NCR+ ILC3 (Lin^-^ NKp46^-^ T-bet^high^ RORγt^+^).

Current literature on human and mouse ILCs has greatly improved our understanding of their function in host immunity. They connect innate and adaptive immune responses by sensing environmental changes, such as infections and inflammation, and by releasing cytokines that regulate immunity. ILCs help maintain T cell immune homeostasis by supporting TH cell differentiation and effector roles. Both ILC2s and ILC3s can internalize and present antigens to TH cells (1, 4, 11). While NK cells and other ILCs have been thoroughly studied in species such as mice, rats, pigs, cattle, and humans, their counterparts in dogs remain poorly understood (12, 13). For example, research on NK cells across species has characterized their features, but the phenotypic characteristics of canine NK cells remain poorly understood and are therefore classified as non-B and non-T large granular lymphocytes (14). Additionally, the phenotypic traits of other canine ILCs are still largely unknown.

Many disorders in humans and animals can alter the numbers, percentages, or functions of leukocytes in the bloodstream (15). Flow cytometry antibody panels are often optimized to identify specific cell lineages, such as ILCs (16). Since ILCs can be mobilized from the blood, migrate between and within organs, and play a role in immune regulation during various inflammatory conditions (17, 18), flow cytometry can be used to phenotypically and functionally analyze them. Therefore, there has been rapid progress in understanding the phenotypes and functions of ILCs in humans and mice in recent years (19), but knowledge about canine ILCs still falls behind that of uncharacterized cells. This gap not only hampers comparative immunology studies but also limits the advancement of translational research in canine models of inflammatory and neoplastic diseases. Given that dogs develop many naturally occurring diseases that closely resemble human conditions, including cancers and immune-mediated disorders, they represent a powerful translational model. Building a solid foundation in this area is crucial for clarifying the roles of ILCs in veterinary diseases and for enabling cross-species insights that inform human immunology and therapeutic development.

## 2 Materials and methods

### 2.1 Isolation of PBMCs

Healthy adult dogs from the Ontario Veterinary College (OVC) community were enrolled in the study. Peripheral blood samples were collected from the jugular vein of healthy dogs into EDTA-containing (purple-top) blood collection tubes and processed for peripheral blood mononuclear cell (PBMC) isolation. Blood collection volumes were based on body weight, with approximately 4 mL collected per 10 kg of body weight, up to a maximum of 20 mL per dog. PBMC isolation and culture procedures were performed under aseptic conditions in a class II biosafety cabinet using sterile reagents and consumables. Culture media were supplemented with 1% penicillin-streptomycin to minimize the risk of microbial contamination. Exclusion criteria included body weight over 25 kg, systemic disease, recent use of systemic immunomodulatory medication or supplements within the preceding two weeks (including corticosteroids and nonsteroidal anti-inflammatory drugs), and vaccination within the past month. Owners were informed about the research project and provided with an informed consent form to sign. Each dog had a complete physical examination, urinalysis, and blood sampling for a complete blood count and serum biochemistry to confirm a healthy status. Follow-up was conducted by email or telephone at one week and six months post-enrollment to ensure no clinically significant illness developed; dogs that became ill were excluded.

Mononuclear cells were isolated from peripheral blood by density gradient centrifugation using Histopaque^®^-1077 (10771-6X, Sigma-Aldrich). Whole blood was diluted 1:1 in phosphate-buffered saline (PBS) (SH3025602, Cytiva) containing 2% heat-inactivated fetal bovine serum (FBS) (35-087-CV, Corning 35-087-CV) and layered onto an equal volume of Histopaque. Samples were centrifuged at 400 × g for 30 min at room temperature with low acceleration and deceleration rates. The PBMC layer was harvested and washed twice in PBS with 10% FBS at 600 × g for 5 min at 4 °C. Cells were resuspended in PBS with 10% FBS, counted with a hemocytometer, and immediately subjected to flow cytometric analysis.

### 2.2 Flow cytometry

PBMCs were analyzed by flow cytometry with a panel of monoclonal antibodies (mAbs) targeting canine-specific or previously published cross-reactive antigens (**Supplementary Table 1**). The total lymphocyte population was defined by gating on CD45^+^ cells using anti−CD45 (clone YKIX716.13, Novus Biologicals). Since no antibodies specific to canine ILC lineages are currently available, we applied a lineage-marker-based exclusion strategy to identify ILCs. Specifically, we gated out T cells, B cells, and myeloid cells using CD3^−^(MA5-16605, Invitrogen), CD5^−^(XJ3723812, Invitrogen), CD21^−^(MA1-19753, Invitrogen), and CD11b^−^(101206, Biolegend). PBMCs were first incubated with a canine Fc receptor (14-9162-42, Invitrogen) (FcR) binding inhibitor polyclonal antibody to prevent nonspecific binding, followed by incubation with a Live/Dead Fixable Dye (423101/423102, BioLegend) to exclude dead cells. Surface marker labeling was performed by incubating PBMCs with the antibody panel. For transcription factor staining (T-bet (25-5825-82, Invitrogen), GATA3 (46-9966-42, Invitrogen) (The clone TWAJ (20, 21) cross-reacts with canine GATA3 (22)), RORγ (ab104950, Abcam)), cells were fixed and permeabilized using the eBioscience™ transcription factor fixation/permeabilization kit buffer (**Supplementary Table 1**). Cells were then resuspended in flow cytometry buffer before being acquired on the Cytek Northern Lights™ flow cytometer in the Department of Pathobiology. At least 70,000 events were acquired and analyzed for each sample. Flow cytometric data were analyzed using FlowJo^®^ software. Fluorescence minus one (FMO) controls were employed to set positive gates for all markers. Gating thresholds were established based on the FMO controls and applied uniformly across all samples and experimental time points.

Following PBMC isolation, the volume of the blood sample was recorded, and the total number of PBMCs recovered was counted using a hemocytometer prior to cryopreservation. Absolute ILC subset numbers were then calculated from the flow cytometry-derived frequencies and the total PBMC yield obtained from a known volume of peripheral blood as follows:


$$\text{PBMCs per mL of the blood}=\frac{\text{Total PBMCs recovered}}{\text{Blood volume collected}}$$

ILC subset cells per mL blood=ILCs subset frequency  determined by flow cytometry×PBMCs per mL blood

### 2.3 *In vitro* expansion cultures of ILCs

ILCs were cultured at 2 × 10^5^ cells/well in DMEM (SH3002201, HyClone) culture medium with 10% FBS + 1% L-Glutamine (25030081, Gibco), 1% penicillin/streptomycin (SV3001, Hyclone), 25 mM HEPES buffer (15630080, Gibco), and 55 μM 2-β-mercaptoethanol (21-985-023, Life Technologies, Carlsbad) in 96-well round-bottom tissue culture plates. Cells were analyzed at defined time points (days 0, 7, 14, and 21) under standard culture conditions (37 °C, 5% CO_2_) to identify the time point of maximal ILC expansion (**Supplementary Data**).

Subsets were stimulated with cytokines as follows: PBMCs were cultured under defined cytokine conditions to expand specific ILC subsets. ILC1 conditions included 50 ng/mL rhIL-2 (PHC0021, Gibco) and 50 ng/mL rcIL-7 (GR122114-10, Genorise); ILC2 conditions included 50 ng/mL rhIL-2, 50 ng/mL rcIL-33 (70005-DNAE-20, Cedarlane), and 50 ng/mL rcIL-7; and ILC3 conditions included 40 ng/mL rh/rcIL-23 (1969-CL-025, Bio-Techne Canada), 50 ng/mL rcIL-7, and 50 ng/mL rhIL-2. The culture medium was replaced every 3 days with freshly prepared, cytokine-containing medium.

## 3 Results and discussion

### 3.1 Results

The current study demonstrates that canine ILCs subsets are detectable in peripheral blood, reliably identified by flow cytometry, and efficiently expanded *ex vivo.* By avoiding the baseline rarity of circulating ILCs, our cytokine-supported cell culture system promoted robust expansion and selective enrichment of all ILC subsets in a donor-dependent manner. In an initial pilot evaluation (**Supplementary Figure 1**), ILC1, ILC2, and ILC3 populations showed a significant increase from day 7 to day 14. While some variability among donors existed, day 14 was the most consistent time point for expansion across all three ILC subsets. Therefore, day 14 was chosen for the follow-up experiments. These complete time−course data are provided in the supplementary figures (**Supplementary Figures 1**, **2**). Based on the consistent peak observed on day 14, this time point was selected as the standardized expansion duration for the subsequent phase of the study, which included 18 dogs. All downstream analyses, therefore, compare freshly isolated cells (day 0) with cells expanded for 14 days under cytokine support.

**Figure 1:**
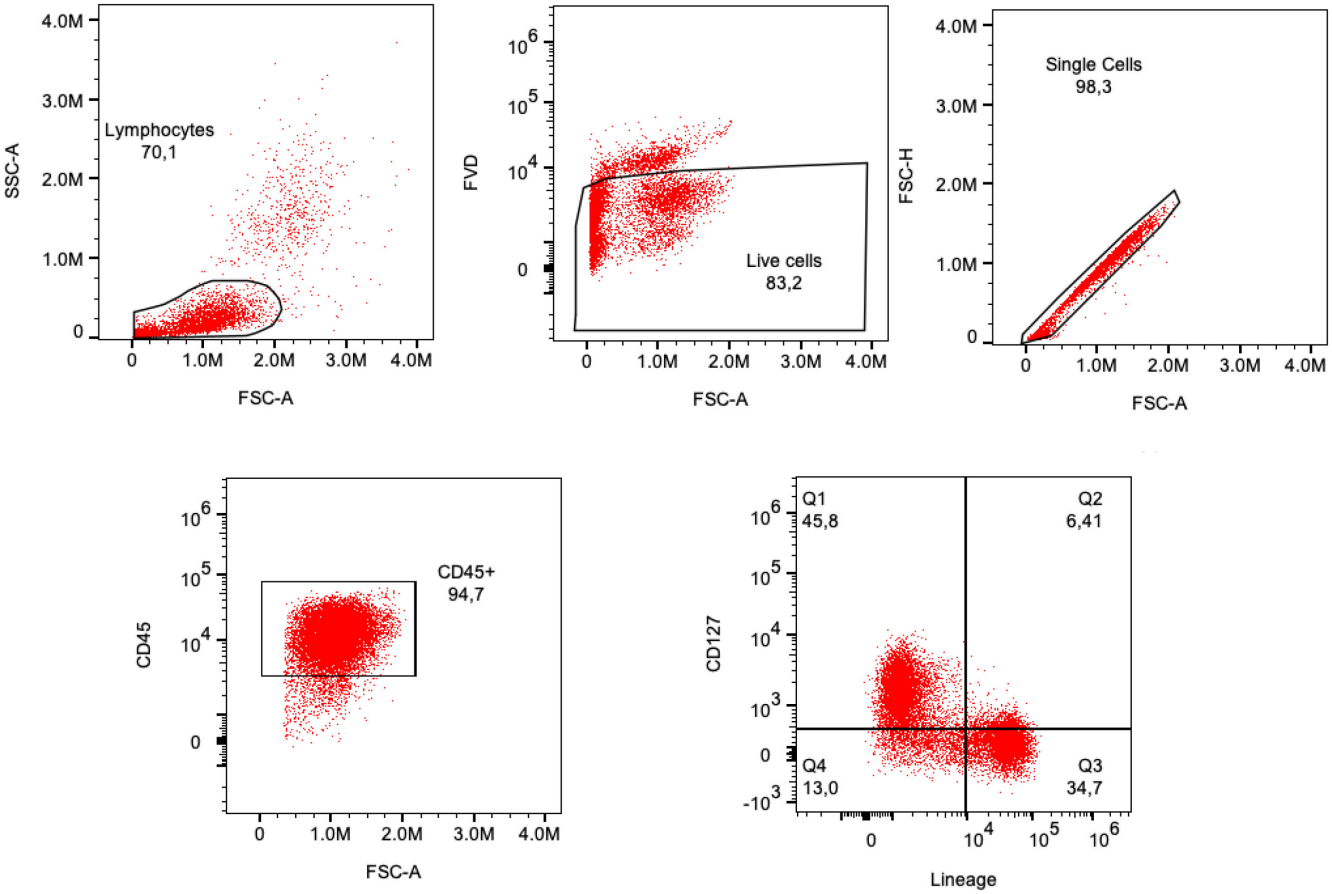
Flow cytometry gating strategy for identifying canine ILC subsets in PBMCs. The plots illustrate the sequential gating strategy used to identify canine ILCs. Cells were first gated on FSC/SSC characteristics to identify the lymphocyte population, followed by live-cell gating and doublet exclusion. CD45^+^ leukocytes were gated, and lineage-positive cells (CD3, CD5, CD21, and CD11b) were excluded. The remaining Lin^−^CD127^+^ population (Q1) was defined as total ILCs and used to identify canine ILC1, ILC2, and ILC3 subsets. Representative flow cytometric dot plots from the *in vitro* expansion of ILC1s are shown.

**Figure 2:**
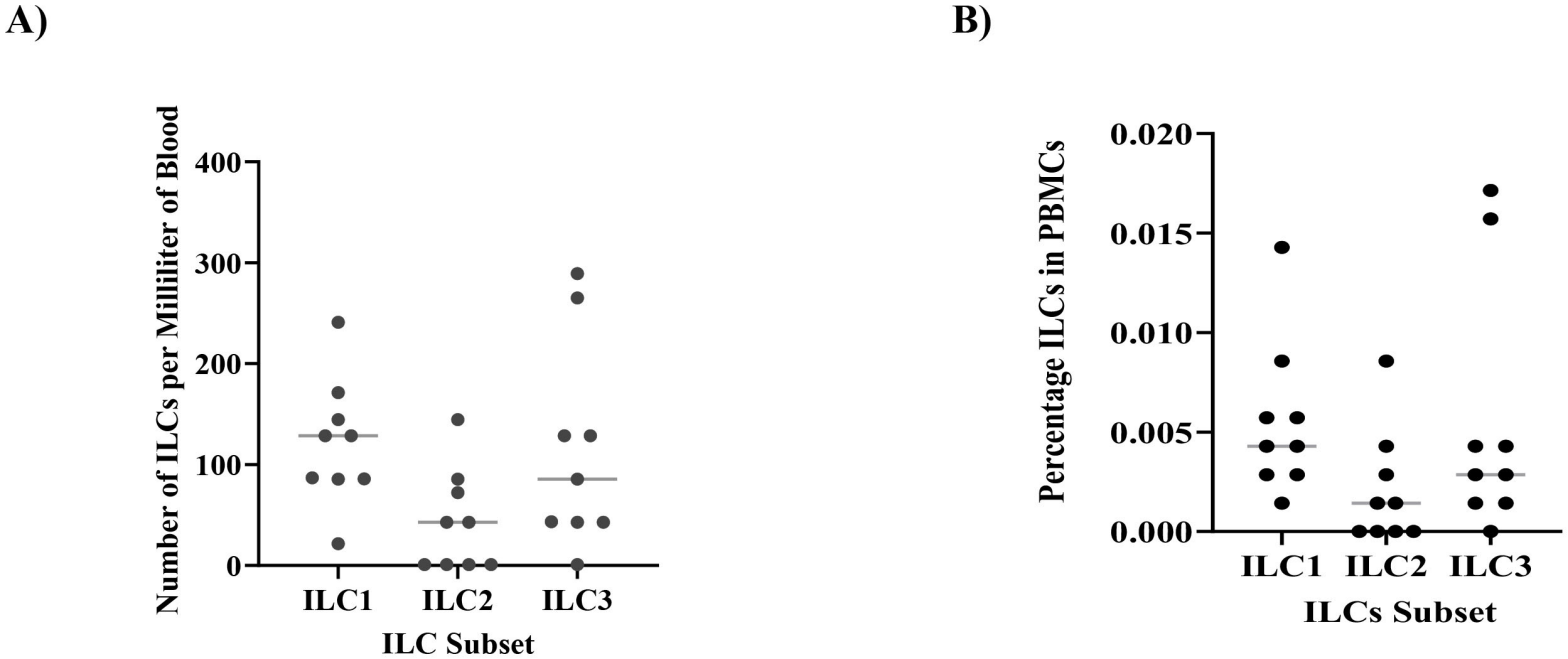
Number of circulating innate lymphoid cell (ILC) subsets per milliliter of peripheral blood in individual dogs. **(A)** Peripheral blood mononuclear cells (PBMCs) were isolated from whole blood collected from nine dogs. ILC subsets were identified as Lin^−^ (CD3^−^CD5^−^CD21^−^CD11b^−^) CD45^+^ cells within the CD127^+^ population, expressing subset-specific transcription factors (T−bet^+^ in ILC1s, GATA−3^+^ in ILC2s, and RORγt^+^ in ILC3s) using flow cytometry. Data are expressed as cells per mL of whole blood. **(B)** Flow cytometry was used to determine the percentages of individual subsets (ILC1, ILC2, and ILC3) within canine PBMCs. Each dot represents an individual dog (n = 9). The Y-axis is shown on a linear scale.

**Figure 3:**
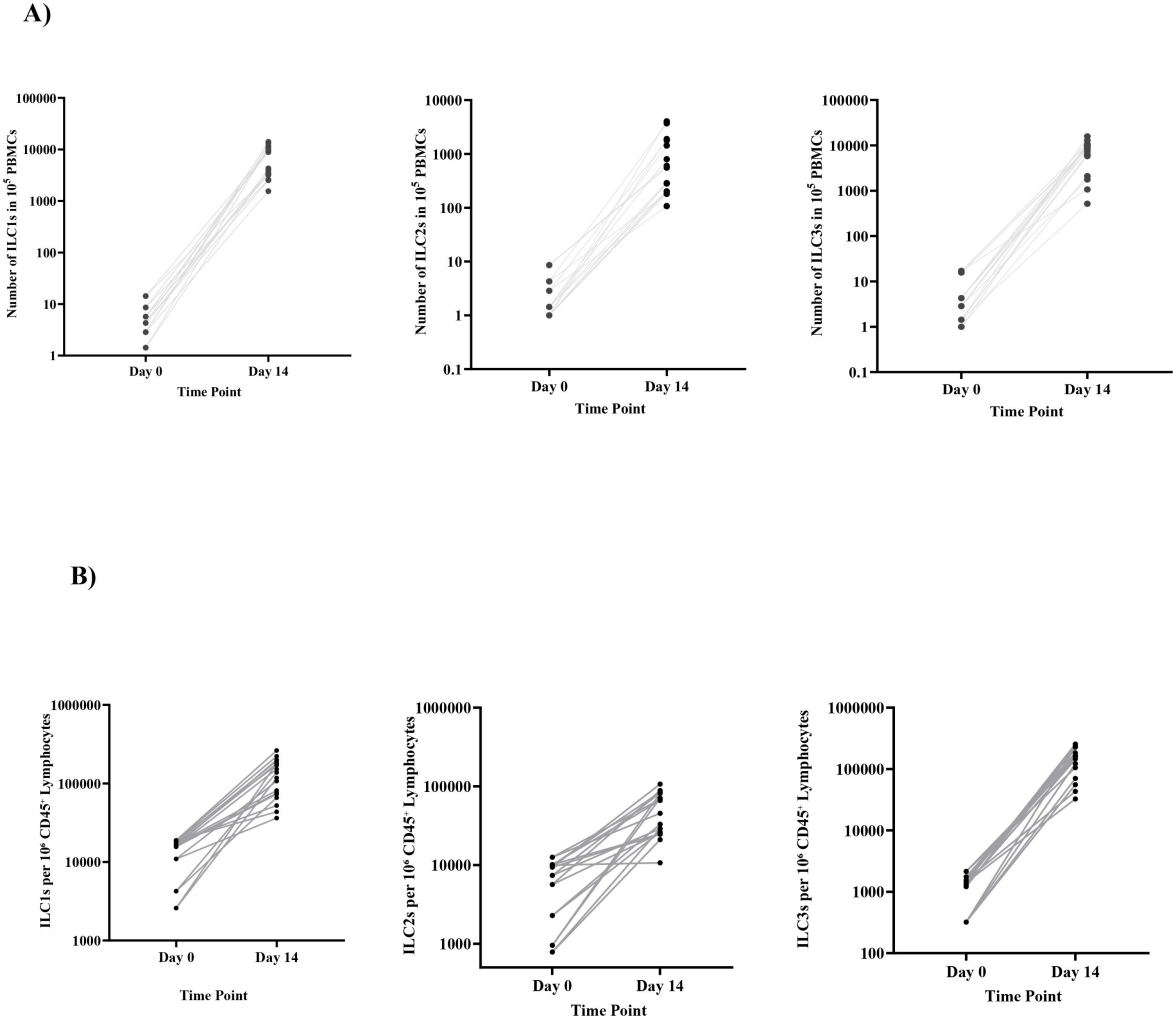
Expansion of ILC Subsets Derived from Canine PBMCs. **(A)** Line plots show total cell counts for ILC1, ILC2, and ILC3 in PBMCs from 9 individual dogs, measured by flow cytometry. 18 samples, which pertain to technical replicates for each donor, were analyzed. ILC subsets were phenotypically defined as CD3^−^CD5^−^CD21^−^CD11b^−^CD45^+^ CD127^+^ lymphocytes that express the subset-specific transcription factors T-bet (ILC1), GATA-3 (ILC2), and RORγt (ILC3). In all subsets, cell numbers were low on day 0 and reached their peak by day 14 under cytokine stimulation. **(B)** The counts of ILC1, ILC2, and ILC3, identified by transcription factor expression, were measured by flow cytometry and reported as cells per 10^6^ CD45^+^ cells.

**Figure 4:**
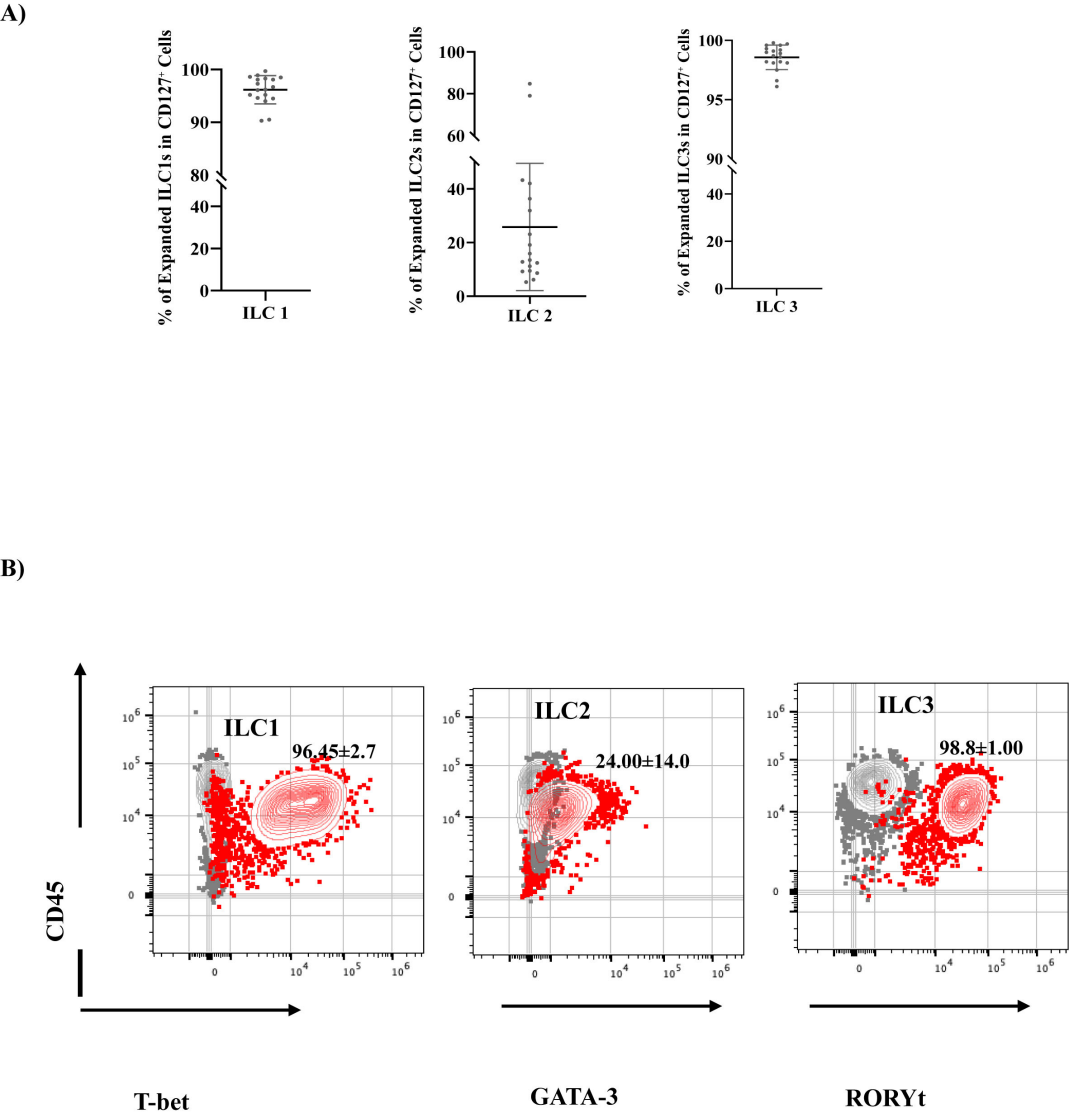
Distribution of ILC subsets after *in vitro* expansion. **(A)** Percentage of ILC subsets within Lin^−^CD45^+^CD127^+^ cells from 9 healthy dogs following 14 days of cytokine-supported expansion culture. Data represent technical replicates for each donor (18 total measurements). **(B)** Representative flow cytometric contour plots of expanded canine ILC1, ILC2, and ILC3 populations. Cells were gated on CD45^+^ lymphocytes and lineage-negative (Lin^−^; CD3^−^, CD5^−^, CD21^−^, CD11b^−^) populations. Within the Lin^−^CD45^+^ compartment, CD127^+^ cells were further analyzed for transcription factor expression to define ILC subsets: T-bet^+^ (ILC1), GATA3^+^ (ILC2), and RORγt^+^ (ILC3). Contour plots show the distributions of these subsets, highlighting clear separation by transcription factor expression (FMO, gray contours; samples, red contours) following *in vitro* expansion. Representative plots are shown from a single donor, with similar patterns observed across independent samples. Percentages of ILCs on the plots are shown as mean ± SD.

Using the gating strategy illustrated in **Figure 1**, canine ILC1, ILC2, and ILC3 populations were identified in peripheral blood, although these subsets were present at extremely low frequencies in freshly isolated PBMCs (**Figures 2A, B**). Following 14 days of cytokine-supported culture, ILC1, ILC2, and ILC3 all increased markedly in both absolute numbers and frequencies relative to freshly isolated PBMCs, with peak expansion consistently observed at this time point across all donors. Although the extent of expansion varied among individuals, all three subsets consistently expanded under the same conditions with different cytokine cocktails (**Figure 3**). Representative flow cytometry dot plots demonstrated clear separation of ILC1, ILC2, and ILC3 populations based on transcription factor expression, illustrating distinct subset identification following expansion (**Figure 4**).

### 3.2 Discussion

In this study, we demonstrate the identification, transcriptional characterization, and *ex vivo* expansion of distinct ILC subsets from canine peripheral blood. While ILC subsets are well-defined in human and murine systems, their identities and functions in dogs remain poorly understood. Our findings demonstrate that canine ILC populations can be reliably resolved using a lineage-exclusion strategy combined with CD127 expression, thereby supporting the identification of distinct ILC subsets. Characterizing ILCs has the potential to advance both veterinary and human immunology. As companion animals, dogs share many environmental exposures with humans and naturally develop a range of pathological conditions, including cancer and immune-mediated diseases. These attributes make dogs valuable models for comparative and translational research. Identifying canine immune cell populations can elucidate conserved immunological mechanisms across species and provide clinically relevant insights that complement and extend findings from conventional experimental systems. A major challenge in canine ILC research is the lack of lineage-specific antibodies validated for flow cytometry. To address this, we adopted an exclusion-based gating strategy, removing T cells, B cells, and myeloid cells (CD3^−^, CD5^−^, CD21^−^, CD11b^−^) prior to identifying ILC subsets. Existing canine immunology literature primarily focuses on NK cells and other innate immune cell types rather than on ILC1, 2, and 3 phenotyping (22, 23). A limitation of this study is that we did not use cytokine production as a criterion for identifying ILC subsets. Functional identification of ILCs traditionally relies on their cytokine profiles (e.g., IFN-γ for ILC1, IL-5/IL-13 for ILC2, and IL-17/IL-22 for ILC3). Although cross-reactive antibodies have been used to detect certain canine cytokines, validated reagents remain limited for comprehensive characterization of canine ILC subsets. Consequently, transcription factor expression was used as the primary approach to distinguish canine ILC populations in this study. The consistent expression of the transcription factors T-bet, GATA-3, and RORγt across expanded populations further supports the classification of canine ILC1, ILC2, and ILC3 subsets. Low percentages of ILC2s expansion, as defined by GATA3 expression, in our expansion coculture system, are another issue we need to investigate further. IL-33 and IL-7 mediate ILC2 development and function. IL-7 induces GATA3 expression, and GATA3 regulates IL-7 receptor expression, creating a regulatory loop vital for ILC2 stability and maintenance (24). IL-33 is a potent driver of GATA3 expression (20), particularly in ILC2s. We included both cytokines in our coculture system. Whether canine GATA3 expression requires additional or canine-specific cytokines or other factors for optimal expression and detection remains to be determined.

Cytokine-driven expansion confirmed that canine ILCs respond to recombinant human IL-2, consistent with its known cross-species activity (22, 25). These findings demonstrate that human cytokines can effectively support canine ILC cultures and highlight the continued limitation in the availability of species-specific reagents. Our findings align with prior studies showing that rhIL-2 supports the growth of canine NK cells, validating its cross-species bioactivity. Importantly, the ability to expand ILC subsets *in vitro* provides a platform for investigating their differentiation and potential roles in immune regulation (26). ILCs have been implicated in tissue homeostasis, mucosal defense, and tumor immunity in humans and mice. The detection and expansion of circulating canine ILCs, along with their close phenotypic similarity to human and mouse ILCs, suggest that these cells may play functionally relevant roles in dogs, a species of growing value in comparative oncology (26, 27).

In our study, the expansion yield of ILC2s is less efficient than that of ILC1s. This could be due to the cell culture system setup, such as the number of ILC2 progenitors in the starting population, the amount of cytokines used, and potential requirements for other factors or cytokines. Additional experiments are required to address these points, including enrichment of the progenitors, testing various concentrations of the cytokines used, conditioning the expansion cell culture system with supportive factors, lineage-specific cytokines, feeder cells, defined extracellular matrices, and metabolic regulators.

In addition, biological heterogeneity among individual dogs should be considered when interpreting the current findings. Although canine ILC populations were consistently identified and expanded across donors, variability was observed between animals, likely reflecting differences in genetic background, age, environmental exposures, and dietary history. Furthermore, the study cohort may not fully capture the diversity of the broader canine population, which may limit the generalizability of the findings. Future studies with larger, more diverse cohorts will be important for validating these observations and further characterizing canine ILC biology.

An important consideration in interpreting our ILC1 population is distinguishing ILC1s from conventional NK (cNK) cells. Recent studies have shown that these populations share several phenotypic and functional characteristics, including expression of T-bet, cytotoxic mediators, and cytokines such as IFN-γ and TNF-α (28). However, ILC1s are generally characterized by the expression of IL-7Rα (CD127) and the absence of lineage markers, including CD3, CD5, and CD11b (29, 30), which are expressed at varying levels across NK cell subsets. Furthermore, canine cNK cells are primarily controlled by transcription factors such as Eomesodermin (Eomes) and T-bet, as well as other factors like LEF1, which are similar to the key regulators of human NK cell development and function (30, 31). In the present study, canine ILC1s were identified as Lin^−^CD127^+^T-bet^+^ cells using currently available canine reagents. Nevertheless, because validated canine markers that definitively distinguish ILC1s from cNK cells are limited, overlap between these populations cannot be completely excluded. Future studies incorporating additional phenotypic and transcriptional markers will be required to further refine the identification of canine ILC1 subsets.

By establishing workflows to identify and expand canine ILC subsets, this study deepens understanding of canine immune biology and provides a foundation for using canine models to bridge basic and human immunology research. Future directions will focus on comprehensive characterization of canine ILCs, including RNA sequencing to define transcriptional profiles and co−culture models to investigate their interactions with tumor cells and pathogens. Such approaches will help clarify the roles of canine ILCs in diseases such as atopic dermatitis and cancer, in which ILC−driven responses are mediated by cell−derived cytokines.

## 4 Conclusion

Our findings show that a specific cytokine-supported culture period is necessary to maximize the enrichment of ILC populations, providing a practical platform for downstream disease modeling applications, including cancer and atopic dermatitis. This system enables analysis of subset-specific effector programs, cytokine responses, and interactions with other immune cells within tumor-associated microenvironments. Additionally, since dogs are increasingly recognized as valuable models for immune-mediated diseases and cancer, the approach described here enables the study of ILC-mediated mechanisms in inflammation, tissue repair, allergy, and tumor immunity. Overall, our results establish a reliable method for isolating and expanding canine ILC subsets from blood, supporting future mechanistic research and the development of ILC-based diagnostic, prognostic, or therapeutic strategies in veterinary and comparative medicine oncology. Importantly, because of the similarities between canine and human immune-mediated diseases, the capacity to resolve and expand canine ILC subsets provides a foundation for using dogs as a translational model to study immune regulation and disease mechanisms.

## Data Availability

The original contributions presented in the study are included in the article/**Supplementary Material**. Further inquiries can be directed to the corresponding author.
