# Supplementary material for: Identification, characterization, and expansion of innate lymphoid cells derived from canine peripheral blood mononuclear cells
Source: Front Immunol. 2026 Jul 15;17:1879697. doi: 10.3389/fimmu.2026.1879697 (PMC13414975; doi:10.3389/fimmu.2026.1879697)
Supplement: Supplementary file 1 [file SupplementaryFile1.docx]

Supplementary Material

**Time‑Course to Determine the Optimal ILC Expansion Day**

To determine the optimal in vitro expansion time point for innate lymphoid cells (ILCs), a pilot time-course study was conducted using samples from four donor dogs (Dogs 1-4). Peripheral blood was collected on day 0, and ILCs were expanded under culture conditions identical for 7, 14, and 21 days. At each time point, cells were harvested and evaluated for total viable cell count and ILC frequency. The goal of this pilot study was to characterize expansion kinetics across the three intervals and identify the most appropriate time point for subsequent experiment analyses.

| Antigen / Reagent | Clone | Species reactivity | Host / Isotype | Catalog # | Source | Fluorochrome | References |
| --- | --- | --- | --- | --- | --- | --- | --- |
| CD45 | YKIX716.13 | Canine | Rat IgG2b | NB100-65906AF405 | Novus Biologicals | Alexa Fluor 405 | N/A |
| CD3ε | CA17.2A12 | Dog | Mouse IgG1 | MA5-16605 | Invitrogen (Thermo Fisher Scientific) | FITC | N/A |
| CD5 | YKIX322.3 | Dog | Rat IgG2a | MA1-81217 | Invitrogen (Thermo Fisher Scientific) | FITC | N/A |
| CD21 | LT21 | Bovine, Dog, Human, Pig | Mouse IgG1 κ | MA1-19753 | Invitrogen (Thermo Fisher Scientific) | FITC | N/A |
| CD11b | M1/70 | Mouse, Human (verified) | Rat IgG2b κ | 101206 | BioLegend | FITC | N/A |
| IL-7Rα (CD127) | R34-34 | Human, Canine | Mouse IgG1 | DDX0700A647-100 | Novus Biologicals | Alexa Fluor 647 | N/A |
| RORγt | 4G419 | Human (Predicted: Dog) | Mouse IgG1 | ab104950 | Abcam | PE | N/A |
| GATA-3 | TWAJ | Human, Mouse, Pig, Rhesus macaque | Rat IgG2b κ | 46-9966-42 | Invitrogen (Thermo Fisher Scientific) | PerCP-eFluor 710 | N/A |
| T-bet | eBio4B10 (4B10) | Human, Mouse, Rhesus monkey | Mouse IgG1 κ | 25-5825-82 | Invitrogen (Thermo Fisher Scientific) | PE-Cy7 | N/A |
| Fc receptor binding inhibitor | Polyclonal | Dog | Polyclonal | 14-9162-42 | eBioscience / Invitrogen | Unconjugated | N/A |
| Fixable viability dye | Zombie Aqua™ | Not applicable | Not applicable | 423101 / 423102 | BioLegend | Zombie Aqua | N/A |

**Supplementary Table 1. Antibody panel used for flow cytometric identification and phenotyping of canine innate lymphoid cells (ILCs) from peripheral blood mononuclear cells** (**PBMCs).** PBMCs were stained with monoclonal antibodies against canine-specific or previously reported cross-reactive surface markers. Total leukocytes were identified by gating on CD45⁺ cells prior to lineage exclusion. A lineage-exclusion strategy (CD3, CD5, CD21, CD11b) was used to define lineage-negative (Lin⁻) ILCs. Intracellular transcription factor staining (T-bet, GATA3, RORγt) was performed following fixation and permeabilization. A canine Fc receptor binding inhibitor and a fixable viability dye were used to minimize nonspecific binding and exclude dead cells.

**Supplementary Results**

**Pilot Study on ILC Expansion Kinetics in Four Dogs**

In vitro expansion of ILCs from PBMCs of 4 dogs showed a consistent increase in ILC numbers from day 0 to day 14, followed by a plateau or decline by day 21. Across all four dogs, day 14 consistently showed the highest fold expansion, with greater ILC frequency and absolute cell counts than at earlier or later time points. Based on the reproducible peak observed on day 14, this time point was selected for subsequent experiments.

| ****  **a** | ****  **b** | ****  **c** |
| --- | --- | --- |

**Supplementary Figure 1. Expansion of ILC subsets derived from canine PBMCs.** Total counts of ILC1, ILC2, and ILC3 from four dogs were measured by flow cytometry on Days 0, 7, 14, and 21 during cytokine-driven PBMC culture (starting input: 2 × 10⁵ cells). Data points represent the means of two technical replicates; lines connect paired donor values. All subsets peaked on Day 14.

|   **a** | |
| --- | --- |
|   **b** |   **c** |

**Supplementary Figure 2. Time-dependent expansion of ILC subsets among CD45⁺ lymphocytes in cultured canine PBMCs.** PBMCs isolated from four individual dogs were analyzed on Days 0, 7, 14, and 21 of cytokine-supported culture. Bars represent the mean of two technical replicate wells ± SEM. The numbers of ILC1, ILC2, and ILC3 were calculated as cells per 10⁶ CD45⁺ lymphocytes, using flow cytometry to determine the frequencies and total numbers of CD45⁺ lymphocytes at each time point.
